# Efficacy and Neural Mechanisms of Mindfulness Meditation Among Adults With Internet Gaming Disorder: A Randomized Clinical Trial

**DOI:** 10.1001/jamanetworkopen.2024.16684

**Published:** 2024-06-18

**Authors:** Haosen Ni, Huabin Wang, Xuefeng Ma, Shuang Li, Chang Liu, Xiaolan Song, Marc N. Potenza, Guang-Heng Dong

**Affiliations:** 1 Department of Psychology, Yunnan Normal University, Kunming, China; 2 Institutes of Psychological Sciences, Hangzhou Normal University, Hangzhou, China; 3 NuanCun Mindful-Living Mindfulness Center, Hangzhou, China; 4 Center of Mindfulness, School of Psychology, Zhejiang Normal University, Jinhua, China; 5 Department of Psychiatry and the Child Study Center, Yale University School of Medicine, New Haven, Connecticut; 6 Department of Neuroscience, Yale University, New Haven, Connecticut; 7 Connecticut Council on Problem Gambling, Wethersfield

## Abstract

**Importance:**

The *Diagnostic and Statistical Manual of Mental Disorders, Fifth Edition, Text Revision* (*DSM-5-TR*), recently identified internet gaming disorder (IGD) as a condition warranting more research, and few empirically validated treatments exist. Mindfulness meditation (MM) has multiple health benefits; however, its efficacy in treating IGD and potential neural mechanisms underlying MM treatment of the disorder remain largely unknown.

**Objective:**

To explore the efficacy of MM used to treat adults with IGD and to identify neural mechanisms underlying MM.

**Design, Setting, and Participants:**

This randomized clinical trial was performed from October 1 to November 30, 2023, at Hangzhou Normal University in Hangzhou, China. Adults (aged ≥18 years) who met at least 6 of the 9 *DSM-5-TR* proposed criteria for IGD were recruited to receive either MM or progressive muscle relaxation (PMR). Data analysis was performed on December 1, 2023.

**Intervention:**

Participants underwent MM training (an 8-session meditation program that focuses on attention and acceptance) and PMR training (an 8-time program for body relaxation) delivered in groups that met 2 times each week for 4 weeks.

**Main Outcomes and Measures:**

This per-protocol analysis included only participants who finished the pretest assessment, 8 training sessions, and posttest assessment. The main outcomes were addiction severity (measured with the *DSM-5-TR* proposed criteria for IGD and with Internet Addiction Test scores), gaming craving (measured with Questionnaire for Gaming Urges scores), and blood oxygen level–dependent signals assessed with cue-craving tasks on fMRI. Behavioral and brain measurements were compared using analysis of variance. Functional connectivity (FC) among identified brain regions was measured to test connectivity changes associated with MM.

**Results:**

This study included 64 adults with IGD. A total of 32 participants received MM (mean [SD] age, 20.3 [1.9] years; 17 women [53%]) and 32 received PMR (mean [SD] age, 20.2 [1.5] years; 16 women [50%]). The severity of IGD decreased in the MM group (pretest vs posttest: mean [SD], 7.0 [1.1] vs 3.6 [0.8]; *P* < .001) and in the PMR group (mean [SD], 7.1 [0.9] vs 6.0 [0.9]; *P* = .04). The MM group had a greater decrease in IGD severity than the PMR group (mean [SD] score change for the MM group vs the PMR group, −3.6 [0.3] vs −1.1 [0.2]; *P* < .001). Mindfulness meditation was associated with decreased brain activation in the bilateral lentiform nuclei (*r* = 0.40; 95% CI, 0.19 to 0.60; *P* = .02), insula (*r* = 0.35; 95% CI, 0.09 to 0.60; *P* = .047), and medial frontal gyrus (MFG; *r* = 0.43; 95% CI, 0.16 to 0.70; *P* = .01). Increased MFG-lentiform FC and decreased craving (pretest vs posttest: mean [SD], 58.8 [15.7] vs 33.6 [12.0]; *t* = −8.66; ƞ^2^ = 0.30; *P* < .001) was observed after MM, and changes in MFG-lentiform FC mediated the relationship between increased mindfulness and decreased craving (mediate effect, −0.17; 95% CI, −0.32 to −0.08; *P* = .03).

**Conclusions and Relevance:**

In this study, MM was more effective in decreasing addiction severity and gaming cravings compared with PMR. These findings indicate that MM may be an effective treatment for IGD and may exert its effects by altering frontopallidal pathways.

**Trial Registration:**

Chinese Clinical Trial Registry Identifier: ChiCTR2300075869

## Key Points

**Question:**

Is mindfulness meditation (MM) an effective treatment for adults with internet gaming disorder (IGD), and what neural mechanisms underlie MM?

**Findings:**

In this randomized clinical trial of 64 participants, IGD severity and craving decreased among participants who received the MM intervention but not those treated with progressive muscle relaxation. The MM intervention was associated with decreased brain activation in the bilateral lentiform nuclei, insula, and medial frontal gyrus.

**Meaning:**

These findings indicate that MM may be an effective treatment for adults with IGD and may exert its effects by altering frontopallidal pathways.

## Teaser

This randomized clinical trial investigates the efficacy and underlying neural mechanisms of mindfulness meditation used to treat adults with internet gaming disorder.

## Introduction

Internet gaming disorder (IGD) has severe negative health effects and has been associated with neurocognitive impairment, executive dysfunction, emotion dysregulation, and physical problems.^1,2,3,4,5^ In the past decade, notable advances have improved understanding of the neural features underlying IGD. Altered brain activation has been observed in frontal brain regions related to executive control.^6^ These regions include the dorsolateral prefrontal cortex and orbitofrontal cortex,^7,8^ which are also implicated in craving. Individuals with IGD may experience strong cravings related to brain activity in other reward processing regions, including subcortical regions such as the striatum or lentiform nuclei.^9,10,11^ Beyond regional activation and consistent with dual-process models of addiction, altered connectivity among brain regions involved in executive control and reward processing have been reported in individuals with IGD.^12,13^ Despite increased understanding of the neurobiology of IGD, treatment development efforts in the behavioral, pharmacologic, and neuromodulatory domains have been arguably slower.^14^ Previous studies have reviewed treatment strategies and their efficacy in treating IGD.^15,16,17,18^ Although treatments are reported to decrease gaming craving or shorten gaming time, gaming recurrence after treatment may be high.^19^

Studies of substance use disorders (SUDs) suggest that craving after cessation is a main reason for relapse.^20,21^ Craving has also been implicated in IGD, and the presence of gaming cues may be difficult to avoid, given how individuals currently use digital devices. Furthermore, games have been used for educational purposes, with schools incorporating games to promote learning.^22,23^ Internet gaming may improve visuospatial ability^24^ and motor skills^25^ and may promote well-being. However, gaming exposure may lead to craving and dysfunction among individuals with IGD.

Mindfulness meditation (MM) has attracted recent public and scientific interest. This treatment has been incorporated into clinical interventions and examined with neuroimaging.^26^ Compared with other treatments, MM has the advantages of versatility and social acceptance, which may increase the likelihood that individuals will engage with and adhere to MM and may overcome some existing barriers to treatment.^27,28^ The practice of MM may extend beyond treating clinical conditions and promote well-being in general populations.^29,30^ To our knowledge, MM has not been systematically investigated to examine its efficacy and tolerability in the treatment of IGD. In MM, individuals are instructed to attend in a nonjudgmental way while maintaining a relaxed vigilance for distractions.^31^ Mindfulness meditation can increase attention and self-regulation,^32^ which often are areas of concern for individuals with IGD. Mindfulness meditation may support sustained improvement by strengthening the ability to monitor and cope with discomfort (craving or negative affect), thus supporting longer-term outcomes.^33^ These characteristics suggest that MM may be beneficial in the treatment of individuals with IGD.

Specific brain mechanisms may underlie the systematic training of attention and self-control with an attitude of acceptance and openness to internal and external experiences inherent to MM.^32,34^ Brain mechanisms implicated in attentional control (alerting, orienting, and conflict monitoring),^35,36^ emotion regulation,^32,34^ and self-awareness (self-reference and awareness of present-moment experiences)^37,38^ have been linked to MM. Specific brain regions implicated include the anterior cingulate cortex, striatum and other limbic regions, prefrontal cortex, insula, posterior cingulate cortex, and precuneus.^32,34^ Thus, MM may alter responses to cues that lead to craving and engagement in addictive behaviors in a manner different from other therapies.^39,40^

Studies of addiction have emphasized a key role for craving.^41,42^ Craving contributes to the development and maintenance of addictive behaviors.^43^ Impaired control over craving has been linked to engagement in addictive behaviors despite adverse consequences. Mindfulness meditation promotes improved attention and self-control^35,38^ and may decrease craving,^44,45^ perhaps by directly altering responses to environmental and internal factors.^44^ Altered activity in the anterior cingulate cortex and prefrontal cortex has been observed in randomized studies of mindfulness training involving individuals who smoke tobacco^46^ or use other drugs.^47^ In previous studies, MM decreased craving and substance use among individuals who used drugs,^37^ with longer-term effects on reduced craving.^33^

Similar to individuals with SUDs, those with IGD often experience impaired executive control over gaming craving^9,10,11^ and alterations in interactions between brain regions involved in executive control and reward processing.^12,13^ The aforementioned findings of efficacy in SUD treatment suggest that MM may be effective in treating individuals with IGD by decreasing cue-related craving and IGD severity. This study aimed to investigate the efficacy of MM in treating adults with IGD and to explore the potential underlying neural mechanisms of MM. Because some prior studies have been criticized for lack of randomization and weak control conditions,^32,34^ we included an active control condition to increase rigor. Based on prior findings for individuals with SUDs, we hypothesized that MM may decrease gaming craving and addiction severity by changing brain mechanisms linked to craving, including decreased activation of cortical and subcortical brain regions and altered interactions among these regions.

## Methods

This randomized clinical trial was approved by the Human Investigations Committee of Hangzhou Normal University in Hangzhou, China. The study was conducted from October 1 to November 30, 2023, and conformed to the World Medical Association International Code of Ethics^48^ and to the principles of the Declaration of Helsinki.^49^ Participants were recruited through advertisements. All participants provided written informed consent. Details of the trial protocol and statistical analysis plan are presented in Supplement 1. This per-protocol analysis included only participants who finished the pretest assessment, 8 training sessions, and posttest assessment. The study followed the Consolidated Standards of Reporting Trials (CONSORT) reporting guideline.

### Participants

To determine the required sample size, we used G*Power, version 3.1.9.7 (University of Dusseldorf), with an effect size *f* equal to 0.25 and α error probability equal to .05. The required sample size was 66. Eligible participants were aged 18 years or older. Potential participants were instructed to complete the online Internet Addiction Test (IAT) (revised for IGD).^50,51^ Individuals with an IAT score greater than 50 were interviewed by a psychiatrist to diagnose IGD according to the proposed criteria in the *Diagnostic and Statistical Manual of Mental Disorders, Fifth Edition, Text Revision* (*DSM-5-TR*), which includes 9 items.^52^ We used a threshold of at least 6 inclusion criteria having been met to ensure IGD of substantial severity.^53^ Psychiatric disorders were also assessed using the Mini-International Neuropsychiatric Interview (MINI).^54^

All participants completed a pretest safety screening questionnaire for functional magnetic resonance imaging (fMRI). Individuals were excluded if they met any of the following criteria: (1) had any nongaming mental or neurological diseases or related histories; (2) had cognitive impairment (indicated with MINI score^54,55^) or depression (indicated with Beck Depression Scale score^56^); (3) had undergone surgery or had head trauma or heart-related diseases in the past year; (4) had claustrophobia; (5) had metal implants and tattoos of the neck or head; (6) had any SUDs during the last 12 months before recruitment; (7) regular used any psychotropic medication; and (8) had any prior mindfulness training experiences.

**Figure 1.**
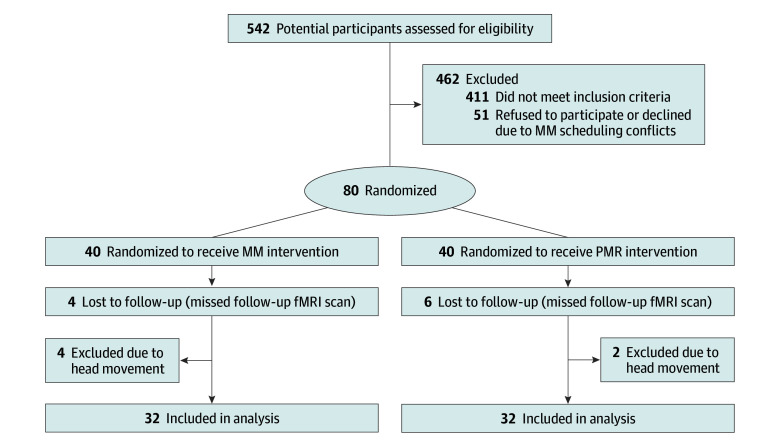
CONSORT Flow Diagram fMRI indicates functional magnetic resonance imaging; MM, mindfulness meditation; and PMR, progressive muscle relaxation.

After careful selection, 80 individuals were included in this study. All eligible participants were randomly allocated (1:1) to either MM (experimental group) or progressive muscle relaxation (PMR) (control group). Men and women were arranged into experimental and control groups separately, according to their registration sequence (odd for experimental and even for control). The CONSORT flow diagram is presented in Figure 1, and the task procedures are described in eFigure 1A in Supplement 2.

### Pretest and Posttest Assessments

#### Behavioral Measurements

Behavioral measurements were collected 1 hour before the pretest assessment. Craving scores for gaming were collected immediately after each MM or PMR session. Data at the posttest assessment were collected immediately after participants finished the eighth MM or PMR session. Addiction severity was measured with the *DSM-5-TR* proposed criteria for IGD and with IAT scores. Craving was measured with the Questionnaire for Gaming Urges (adapted from the Tiffany Questionnaire for Smoking Urges^57^) before the first training, after each training, and 1 month after the entire training period.

#### Task-Based Brain Responses

Participants performed a cue-craving task during fMRI as described previously.^58^ eFigure 1B in Supplement 2 describes the tasks and timeline for 1 trial. First, participants were asked to fixate their sight on a cross located at the center of the screen for 500 milliseconds. Then cues were presented for approximately 3000 milliseconds, and participants were instructed to respond to whether there was a face in each picture by pressing button 1 (yes) or 2 (no). Each cue was terminated by pressing a button. If participants did not respond for 3000 milliseconds, the trial was considered missed. After participants pressed the button, a black screen was presented for 3000 milliseconds. Finally, a black screen was shown for 1500 to 3500 milliseconds before the next trial. The task included 80 trials, with the entire task lasting approximately 12 minutes.

Of the 80 pictures shown in the task, 40 were related to gaming and 40 were related to typing (eFigure 1B in Supplement 2). In each category, half of the pictures had a face and the other half had a hand. In gaming-related pictures, a person was shown gaming in front of a computer. Typing-related pictures were considered neutral stimuli. For our study, we created 2 copies (A and B) of the tasks for pretest and posttest. To avoid having a difference between A and B (although we tried to make them similar), some of the participants used copy A for the pretest and copy B for the posttest; the other participants used copy B for the pretest and copy A for the posttest. This is known as the AB/BA experimental design.

### Mindfulness Training Sessions

All MM and PMR training sessions were performed in separate rooms, in which all participants had a seat or yoga mat. Beyond MM and PMR training, no specific requirements were set for participants regarding their gaming behaviors. The interventions are described next.

eFigure 1 in Supplement 2 presents the MM (experimental) and PMR (control) training interventions. Training lasted for 4 weeks, with 2 sessions (each 2.5-3.5 hours) per week. We required participants to attend all training and fMRI scanning sessions.

The MM training sessions typically included 10 to 20 participants and were led by 2 MM trainers. (The PMR training sessions were open at the same time and were led by 2 PMR trainers.) During the training sessions, trainers observed participants and answered questions after training completion. The training procedure’s design was based on specified theoretical content.^59^ Details are provided in the eAppendix in Supplement 2.

Participants in the control group attended the same number and length of group PMR sessions and also received information about body relaxation. We considered several methods, including group intervention, group lessons, and training camps, when selecting activities for the control group. After careful consideration, we believed that PMR would be most appropriate. Progressive muscle relaxation, as proposed by Edmund Jacobson, MD, PhD, has demonstrated efficacy in decreasing anxiety and stress and improving sleep.^60,61^ Detailed reasons for selecting PMR and steps of the PMR intervention are presented in the eAppendix in Supplement 2.

### Parameters and Preprocessing of fMRI Scans

Structural images were obtained using a T1-weighted, 3-dimensional, spoiled gradient-recalled sequence covering the whole brain (176 slices; repetition time, 1700 milliseconds; echo time, 3.93 milliseconds; slice thickness, 1.0 mm; skip, 0 mm; flip angle, 15°; inversion time, 1100 milliseconds; field of view, 240 × 240 mm^2^; and in-plane resolution, 256 × 256). We performed fMRI using a Sigma 3T scanner (GE HealthCare) with a gradient echo-planar imaging, T2-weighted, sensitive-pulse sequence in 33 slices (interleaved sequence; thickness, 3 mm; repetition time, 2000 milliseconds; echo time, 30 milliseconds; flip angle, 90°; field of view, 220 × 220 mm^2^; and matrix, 64 × 64).

Functional volumes were slice-time corrected and realigned using the Statistical Parametric Mapping package, version 12 (Functional Imaging Laboratory, UCL Queen Square Institute of Neurology),^62^ then co-registered and normalized to the Montreal Neurological Institute brain template and smoothed using a 4-mm^3^ isotropic gaussian kernel. Six participants (4 in the MM group and 2 in the PMR group) were excluded from analyses due to head movement (3 mm in directional movement or 2° in rotational movement). Detailed procedures and parameters are provided in the eAppendix in Supplement 2.

#### Primary Analysis

A general linear model (GLM) analysis was performed to identify blood oxygen level–dependent (BOLD) activation using NeuroElf, version 1.1, a pipeline data processing tool based on Statistical Parametric Mapping and BrainVoyager, generated by Jochen Weber (Sleep, Cognition and Neuroimaging Laboratory, Columbia University).^63^ Different types of trials (gaming pretest, neutral pretest, gaming posttest, and neutral posttest) were separately convolved with a canonical hemodynamic response function for task regression. The duration of each trial was 2000 milliseconds, and GLMs included a constant term per run. Head movement parameters and a high-pass filter (0.01-0.1 Hz) for 128 seconds were included as regressions of no interest. The GLM approach was used to identify voxels that were significantly activated for each event during the response stage.

#### Secondary Analysis

First, we identified voxels that showed a main effect in the gaming trials compared with the neutral trials. Second, we determined voxels that were significantly different in pretest vs posttest BOLD signals. We identified clusters of contiguous and significantly different voxels at an uncorrected threshold of *P* < .001 (2-sided). Finally, these clusters were tested for cluster-level familywise error correction at *P* < .005 (2-sided). Specifically, the estimate obtained using the AlphaSim correction method indicated that the cluster extent of 26 adjoining voxels would achieve a familywise error threshold of *P* < .005 (2-sided) effectively. The smoothing kernel applied in simulating false-positive (noise) maps using AlphaSim was 8.4 mm, which was estimated from residual fields of the contrast maps pooled into the 1-sample *t* test.

### Statistical Analysis

#### Behavioral Measurements and fMRI Analysis

Analysis of variance (ANOVA) of group (MM or PMR) × time (pretest [baseline] or posttest [outcome]) interactions were performed. Post hoc analyses were performed for further testing. *P* < .05 was considered statistically significant, and all significance tests were 2 tailed.

#### Functional Connectivity Among Implicated Brain Regions

We used brain regions implicated in the cue-craving task as regions of interest (ROIs) in group comparisons and calculated the functional connectivity (FC) among these ROIs. Correlations between pretest vs posttest changes in FC values and changes in gaming craving scores were calculated.

Statistical analysis was performed using the CONN toolbox, release 22a.^64^ Data analysis was conducted on December 1, 2023.

## Results

Of the 80 adults with IGD recruited for this trial, 16 did not complete the 2 pretest and posttest fMRI scans and 8 MM or PMR training sessions. Therefore, our analysis included 64 participants. A total of 32 participants received the MM intervention (mean [SD] age, 20.3 [1.9] years; 17 women [53%] and 15 men [47%]) and 32 received the PMR intervention (mean [SD] age, 20.2 [1.5] years; 16 women [50%] and 16 men [50%]). Demographic characteristics of both groups are presented in the Table.

**Table.** Demographic Characteristics of Participants

Characteristic	Intervention (N = 64)	
	MM (n = 32)	PMR (n = 32)
Sex, No. (%)		
Male	15 (47)	16 (50)
Female	17 (53)	16 (50)
Age, mean (SD), y	20.3 (1.9)	20.2 (1.5)
Assessment score, mean (SD)		
IAT	70.1 (10.1)	69.3 (9.7)
*DSM-5-TR* proposed criteria for IGD	7.0 (1.1)	7.1 (0.9)
QGU	58. 8 (15.7)	57.4 (12.1)

Abbreviations: *DSM-5-TR*, *Diagnostic and Statistical Manual of Mental Disorders, Fifth Edition, Text Revision*; IAT, Internet Addiction Test (revised version for IGD); IGD, internet gaming disorder; MM, mindfulness meditation; PMR, progressive muscle relaxation; QGU, Questionnaire for Gaming Urges.

### Behavioral Measures

**Figure 2.**
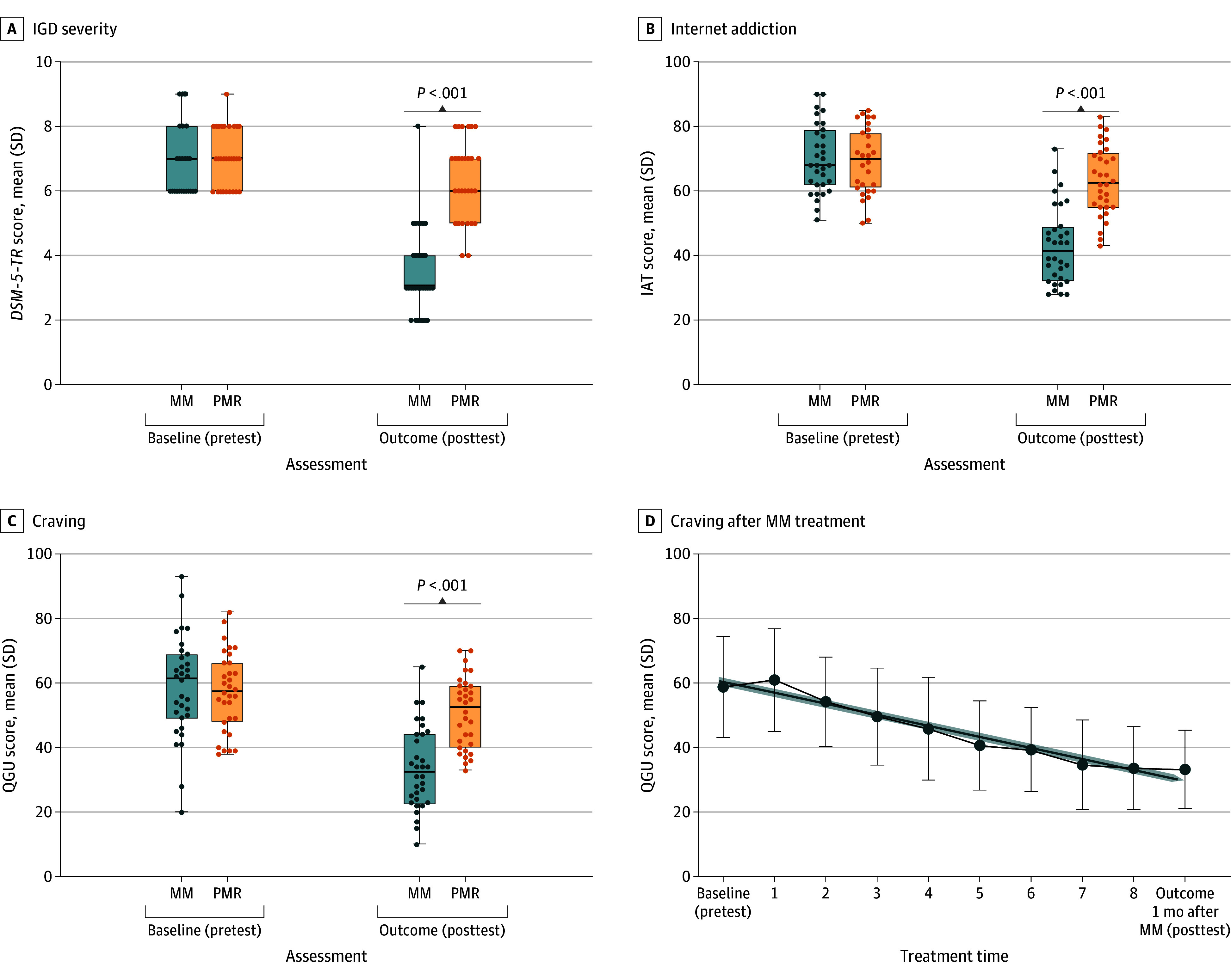
Pretest and Posttest Assessments of Mindfulness Meditation (MM) and Progressive Muscle Relaxation (PMR) A to D, Baseline (pretest) and outcome (posttest) assessments of internet gaming disorder (IGD) severity according to the number of *Diagnostic and Statistical Manual of Mental Disorders, Fifth Edition, Text Revision* (*DSM-5-TR*) proposed criteria met (A); internet addiction, assessed with Internet Addiction Test (IAT) scores (B); and craving overall (C) and after each MM treatment (D), assessed with Questionnaire for Gaming Urges (QGU) scores. A-C, Solid circles indicate individual participant scores; horizontal lines, mean value; boxes, range between 75% and 25% of participants; and whiskers, range between maximum and minimum values. D, Solid circles indicate the mean value of each measure; whiskers, the range; solid black line, the link among the values; and shaded line, the trend of these values. *P* values compare the posttest results between the MM and PMR groups.

Statistically significant effects of group (MM or PMR) × time (posttest or pretest) interactions were observed for the *DSM-5-TR* proposed criteria for IGD. The MM group had a significant decrease in the number of *DSM-5-TR* proposed criteria met (pretest vs posttest: mean [SD], 7.0 [1.1] vs 3.6 [0.8]; *P* < .001). The PMR group also had a significant decrease in the number of *DSM-5-TR* proposed criteria met (pretest vs posttest: mean [SD], 7.1 [0.9] vs 6.0 [0.9]; *P* = .04) (Figure 2A).

Similar findings were observed for IAT scores (Figure 2B) and craving (Figure 2C) for the MM group but not for the PMR group. In the MM group, there were significant decreases in IAT scores (pretest vs posttest: mean [SD], 70.1 [10.1] vs 43.0 [12.0]; *P* < .001) and craving scores (mean [SD], 58.8 [15.7] vs 33.6 [12.0]; *t* = −8.66; ƞ^2^ = 0.30; *P* < .001). In the PMR group, decreases in IAT scores (pretest vs posttest: mean [SD], 69.3 [9.7] vs 63.2 [10.7]; *P* = .11) and craving scores (mean [SD], 57.4 [12.1] vs 50.9 [11.0]; *P* = .14) were not statistically significant (eFigures 2 and 3 in Supplement 2).

When posttest results were compared between the MM and PMR groups, statistically significant differences were found between groups for IGD severity (mean [SD], 3.6 [0.8] vs 6.0 [0.9]; *P* < .001), IAT scores (mean [SD], 43.0 [12.0] vs 63.2 [10.7]; *P* < .001), and craving scores (mean [SD], 33.6 [12.0] vs 50.9 [11.0]; *P* < .001) (Figure 2, A-C). The MM group had a greater decrease in IGD severity than the PMR group (mean [SD] score change, −3.6 [0.3] vs −1.1 [0.2]; *P* < .001). Figure 2D presents changes in craving scores for the MM group from pretest assessment at baseline to 1 month after posttest assessment.

### Task-Based Brain Responses

**Figure 3.**
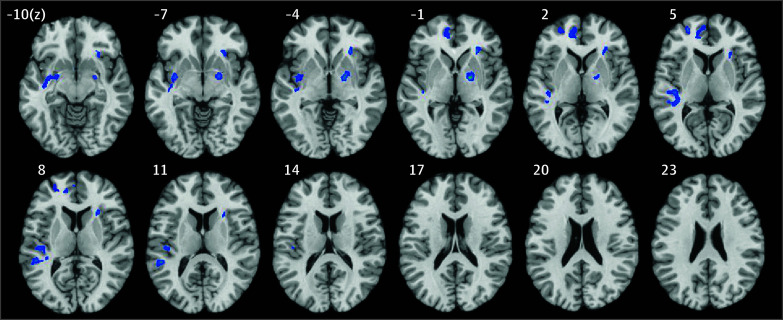
Brain Response Changes Associated With Mindfulness Meditation (MM) and Progressive Muscle Relaxation (PMR) Training After MM, decreased brain responses were observed in the bilateral lentiform nuclei, left medial frontal gyrus, right insula, and right sublobar region. *Z* refers to the superior (+) or inferior (−) of the Montreal Neurological Institute brain coordinate. The blue markings indicate the areas that showed decreased brain activity in the group (MM or PMR) × time (posttest or pretest) analyses.

The ANOVA results showed that brain responses in the bilateral lentiform nuclei, left medial frontal gyrus (MFG), right insula, and right sublobar region decreased after MM treatment compared with baseline, with greater effects than PMR (Figure 3 and eTable in Supplement 2). In the comparison of β weights in different conditions, these features were related to decreased brain responses after MM treatment (Figure 3). Regarding relationships between pretest vs posttest changes in brain response and craving scores, positive correlations were observed in the lentiform nuclei (*r* = 0.40; 95% CI, 0.19 to 0.60; *P* = .02), insula (*r* = 0.35; 95% CI, 0.09 to 0.60; *P* = .047), and MFG (*r* = 0.43; 95% CI, 0.16 to 0.70; *P* = .01) (Figure 3). Findings for the fusiform and sublobar regions are presented in eFigure 4 in Supplement 2.

### FC Among Implicated Brain Regions

**Figure 4.**
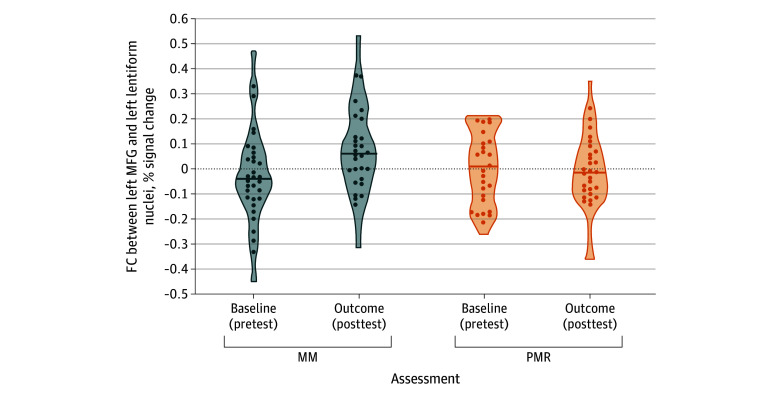
Functional Connectivity (FC) Between the Mindfulness Meditation (MM) and Progressive Muscle Relaxation (PMR) Groups Solid circles indicate individual participant values; horizontal lines, the mean value among participants in each group. MFG indicates medial frontal gyrus.

eFigure 5 in Supplement 2 describes the ROIs selected for FC calculation. Functional connectivity between the left MFG and left lentiform increased after MM (Figure 4). Changes in MFG-lentiform FC mediated the relationship between changes in mindfulness and gaming craving scores in the MM group (task A-B = −0.17; 95% CI, −0.32 to −0.08; *P* = .03; eFigure 6 in Supplement 2).

## Discussion

In this study of adults with IGD, MM was superior to PMR in reducing craving and IGD severity. Furthermore, frontopallidal brain mechanisms were implicated, with changes in MFG-lentiform FC mediating the relationship between increased mindfulness and decreased craving. Implications of these findings are discussed next.

### Pretest vs Posttest Assessments

Most pretest vs posttest indicators of IGD used in this study (ie, IAT scores, number of *DSM-5-TR* proposed criteria met, and craving scores) decreased in both the MM and PMR groups. These findings highlight the importance of including a control PMR group to identify effects specific to MM. The ANOVA of group (MM or PMR) × time (pretest or posttest) interactions indicated superiority of MM on outcome measures related to craving and IGD severity. The results suggest that 8 MM training sessions reduced gaming craving to a significantly greater extent than PMR, supporting the promise of MM in treating individuals with IGD.

### Brain Responses

According to our group comparisons, MM decreased activation in brain regions previously implicated in craving, including the lentiform nuclei, MFG, and insula. The lentiform nucleus is a key node of dopaminergic circuitry implicated in craving and reward processing.^65,66^ Cue-induced craving often involves lentiform activation, including in individuals with IGD^42,67^ and those with SUDs.^68,69^ Recovery and decreased addiction severity have been associated with decreased lentiform activation in cue-craving tasks.^67^ In line with these prior reports, positive correlations between gaming craving and lentiform activation were observed in this study, with decreased activation after MM treatment. Taken together, cue-related lentiform activation may represent a potential biomarker of treatment outcome for IGD, with MM serving to decrease such activation.

In this study, the MFG also demonstrated decreased cue-related activation after MM. In a previous meta-analysis, MFG activation was implicated in cue-induced drug craving.^70^ The MFG shows temporal dynamics in drug-cue responses^71^ and is also implicated in executive control.^65,72^ In general, enhanced brain reactivity of these areas to relevant addictive cues suggests that individuals may be attempting to inhibit urges in the presence of cues^66^ or, perhaps more likely, that prefrontal regions are involved in craving circuitry, consistent with network-based analyses.^73,74,75^ Mindfulness meditation may operate, in part, by altering cue reactivity and thus increasing behavioral control.^32,34^

The features of MM linked to cue responsivity warrant consideration. Mindfulness meditation involves systematic training of attention and self-control with an attitude of acceptance and openness to internal and external experiences.^32,34^ One key feature of MM involves accepting the present situation in a nonjudgmental fashion while maintaining a relaxed vigilance for distractions.^31^ In the case of craving, MM may involve considering cravings or urges as current phenomena, observing them with curiosity as mental events, and not suppressing them or thinking about the past or the future.^76^ With respect to IGD, MM may involve accepting gaming cravings and not engaging in gaming or combatting the urges. Previous randomized MM studies have observed reduced resting brain activity in cortical regions of individuals with tobacco use disorder^46^ or other SUDs,^47^ consistent with findings that individuals who are experienced in meditation show relatively reduced activity in cortical regions, especially in regions of the default mode network such as the MFG and posterior cingulate cortex.^37^ As such, the brain mechanisms underlying MM may differ from those of other behavioral treatments such as cognitive behavior therapy or motivational interviewing, although some common elements (eg, decreased neural craving responses to cues) may be shared.^77,78^

In this study, decreased insula activation was observed after MM in the experimental group during the fMRI craving task. The insula, like the lentiform nuclei and cortical regions, has been implicated in craving^79,80^ and has thus been proposed as a treatment target for addiction.^81^ Nicotine withdrawal has been associated with greater activation of the anterior insula,^82^ and individuals with stroke lesions in the insula have spontaneously quit smoking.^83,84^ The insula has been linked to reactivity to gaming cues. However, among individuals with IGD, relatively increased activation to gaming cues was observed after a craving behavioral intervention (involving elements of mindfulness and cognitive behavior therapy), although decreased connectivity between the insula and regions implicated in craving like the precuneus was also observed.^85,86^ Additionally, relatively decreased insula activity has been observed in individuals with IGD in response to affective stimuli.^87^ From this perspective, the insula has been implicated in complex or mixed functions in IGD. In this study, the correlation between changes in insula activation and gaming craving scores suggests that MM may reduce craving in part by reducing insular activation to gaming cues, similar to observations for SUDs.

### MM and Changes in FC

In this study, both the MFG and lentiform nuclei showed relatively decreased cue-related activity after MM, with increased MFG-lentiform FC. These findings suggest that concurrent reductions within the frontopallidal circuitry may be related to decreased cue reactivity and craving after MM. Consistent with this notion, we observed that FC changes between the MFG and lentiform nuclei mediated the relationship between changes in mindfulness and gaming craving. As discussed earlier, FC between the MFG and lentiform nuclei reflects the coupling between these 2 measures. Addiction may involve an imbalance between control and reward systems, which may be structurally independent but functionally coordinated^88^; in addition, top-down regulation of craving in addiction may involve cortical control over subcortical drives.^89^ Although the results for MM in this study suggest such processes, a perhaps more plausible explanation given the fMRI task administered suggests that MM may directly alter cortical-subcortical systems underlying craving. Future studies involving different tasks, such as regulation-of-craving tasks,^72^ particularly those modified to examine mindful vs cognitive regulation, may help provide further clarity.

### Limitations

This study had several limitations. First, this study included only gaming cue task–based brain responses. Data from other tasks or resting-state data may have provided additional insight. Second, the pretest and posttest assessments were not linked to specific MM modules. Future studies may consider more precisely the elements of MM that may reflect its active ingredients. Third, follow-up of these participants would provide valuable information about the recurrence of gaming addiction after MM training.

## Conclusions

In this randomized clinical trial of adults with IGD, MM was more effective in decreasing addiction severity and gaming cravings compared with PMR. In addition, MM decreased activation in cortical and subcortical brain regions previously implicated in craving responses, with coordinated reductions linking relationships between changes in mindfulness and decreases in cue-induced craving. These findings suggest that MM may alter frontopallidal responses underlying craving in IGD, and they provide preliminary support for the use of MM to treat IGD and suggest possible neural mechanisms for its efficacy.

## Notes

Supplement 1.
Trial Protocol

Supplement 2. **eAppendix.** Details for Mindfulness Meditation (MM) and Progressive Muscle Relaxation (PMR) Training Used in the Current Study **eFigure 1.** Timeline for Treatment and Assessments **eFigure 2.** Correlation Between Brain Response Changes in the Fusiform Gyrus (FG) and Craving Score Changes **eFigure 3.** Correlation Between Brain Response Changes in the Sublobar Region and Craving Score Changes **eTable.** Brain Responses Between MM and PMR Groups at Posttreatment and Pretreatment (Analysis of Variance) **eFigure 4.** Changes in Brain Responses Associated With MM and PMR **eFigure 5.** Regions of Interest Selected for Further Analysis **eFigure 6.** Functional Connectivity (FC) Changes in Medial Frontal Gyrus (MFG)-Lentiform Nuclei Mediate the Association Between Changes in Mindfulness and Craving Scores (Posttreatment to Pretreatment) in the MM Group

Supplement 3.
Data Sharing Statement
