# Supplementary material for: Efficacy and Neural Mechanisms of Mindfulness Meditation Among Adults With Internet Gaming Disorder: A Randomized Clinical Trial
Source: JAMA Netw Open. 2024 Jun 18;7(6):e2416684. doi: 10.1001/jamanetworkopen.2024.16684 (PMC11185988; doi:10.1001/jamanetworkopen.2024.16684)
Supplement: Supplement 1. — Trial Protocol [file jamanetwopen-e2416684-s001.pdf]

Protocol Title:

Efficacy and Neural Mechanisms of Mindfulness Meditation Among Adults With Internet Gaming Disorder: A Randomized Clinical Trial

Protocol Number (Clinical Trial Registration Identifier):

The protocol trial was registered at the Chinese clinical trial registry (www.chictr.org.cn; ChiCTR2300075869).

Ethics and dissemination: The study was approved bythe ethic committee of human research at Yunnan University in China. The experimental procedure was in accordance with the ethical principles of the 1964 Declaration of Helsinki (World Medical Organization, 1996).

Name of funding source or sponsor: This research was supported by Zhejiang Provincial Natural Science Foundation (LY20C090005).

Purpose of Project and Scientific Justification

As internet gaming disorder (IGD) was introduced in the DSM-5, few empirically validated treatments exist. Mindfulness mediation (MM) has multiple health benefits. However, its efficacy in treating IGD and the potential neural mechanisms underlying MM treatment of IGD remain largely unknown.

Purpose: The current study aimed to investigate the efficacy of MM in helping people with IGD and to explore the potential underlying neural mechanisms. Based on prior findings in SUDs, we hypothesized that MM could decrease gaming craving and addiction severity by changing brain mechanisms linked to craving, including decreased activation of cortical and subcortical brain regions and altered interactions among these regions.

Criteria for Patient Selection

Potential participants were instructed to complete the online internet addiction test (IAT; www.netaddiction.net) (Widyanto et al., 2011; Widyanto & Mcmurran, 2004). Individuals scoring higher than 50 were interviewed by a psychiatrist to diagnose IGD according to the DSM-5 criteria (American Psychiatric Association, 2013). We used a threshold of at least 6 inclusionary criteria having been met to ensure significant IGD (G. H. Dong et al., 2020). Psychiatric disorders (through the use of the Mini-International Neuropsychiatric Interview (MINI)) were also assessed (Y Lecrubier et al., 1997).

All participants completed a safety screening questionnaire for fMRI. Individuals were excluded for the following: (1) Any non-gaming mental or neurological diseases or related histories; (2) Cognitive impairment on the MINI (Yves Lecrubier et al., 1997; Sheehan et al., 1998) or depression on the Beck Depression Scale (Beck et al., 1967); (3) Surgery, head trauma, or heart-related diseases in the past year; (4) Claustrophobia; (5) Metal implants and tattoos of the neck or head; (6) Any SUDs during the last 12 months before recruitment; (7) Use of any psychotropic medication regularly; and, (8) Any prior mindfulness training experiences.

Groups

All eligible participants were randomly allocated to two groups: mindfulness meditation (MM) and a progressive muscle relaxation (PMR).

After careful selection, 80 subjects were included in the current study. They were randomly assigned to an experimental or control group (40:40). Sixteen subjects did not complete the two scans and 8 MM/PMR training classes, leaving 64 (32:32) subjects finishing all experimental procedures.

Main measures

Behavioral measures (before and after mindfulness training)

Addiction severities were measured through the DSM-5 proposed criteria and Young's IAT. Cravings were measured by a revised questionnaire for gaming (adapted from the Tiffany Questionnaire for Smoking Urges) before first training, after each training, and one month after the entire training period. Mindfulness was measured through the Five Facet Mindfulness Questionnaire (FFMQ).

Cue-craving task in the scanner (before and after training)

Subjects performed a cue-craving task during fMRI as described previously (W. R. Zhou et al., 2021). Figure 1B shows the task procedures and the timeline for one trial of the task. First, subjects were asked to fixate their sight on a cross located at the center of the screen for 500 ms. Then, cues were presented for approximately 3000 ms, and subjects were instructed to respond to whether there was a face in each picture by pressing buttons "1" (yes) or "2" (no). Each cue was terminated by pressing a button. If subjects did not respond for 3000 ms, the trial was considered a missed trial. After pressing the button, a black screen was presented for 3000 ms. Finally, a black screen was shown for 1500-3500 ms before the next trial. The task included 80 trials, with the entire task lasting approximately 12 min.

Of the 80 pictures shown in the task, 40 were related to gaming and 40 were related to typing (Figure 1B right). In each category, half pictures had a face, and the other half had a hand. In gaming-related pictures, a person was shown gaming in front of a computer. Typing-related pictures were considered neutral stimuli. We created two copies (copy A/B) of this task, and the two were counterbalanced across different subjects (A-B or B-A).

Consent Documentation

All the subjects participated in the study voluntarily and signed a written informed consent. The study was approved by local ethic committee of human research at hangzhou normal University in China.

Alternatives to Participation

Subjects either do not receive any treatment in this study or forego treatment in order to participate in this study. The alternative, therefore, is not to participate.

被 试 知 情 同 意 书

(RESEARCH CONSENT FORM)

姓名 name		性别 gender		出生日期 Birth	
身高		体重		受教育程度	
国籍		身份证号			

课题题目<br>(Project Title)	正念冥想训练对游戏成瘾的干预效果及其机制<br>Mindfulness meditation training on IGD and its cognitive mechanisms

身份<br>(Identity)	姓名 职称	研究单位	
主要研究者<br>(Principal Investigator)	董光恒 教授	云南师范大学 教育学部心理系	
合作研究者<br>(Co-Investigator)	倪浩森 （学生）	云南师范大学 教育学部心理系	
合作研究者<br>(Co-Investigator)	马雪峰 （学生）	云南师范大学 教育学部心理系	
合作研究者<br>(Co-Investigator)	李爽（学生）	云南师范大学 教育学部心理系	
合作研究者<br>(Co-Investigator)			
合作研究者<br>(Co-Investigator)			
合作研究者<br>(Co-Investigator)			
被试类型<br>(Subject Population)	健康志愿者 (Healthy volunteer)		
研究联系人<br>(Study Contact)	董光恒 Guangheng Dong		
联系电话<br>(Telephone)	15867949909	**Email:**	Dongguangheng@ynnu.e

研究目的(Purpose)

正念冥想在目前获得科学界的高度重视。因为其易于接受，作用广泛等特点在临床精神疾病干预中广泛应用。我们的研究计划采用正念冥想的策略对游戏成瘾的人群进行干预，正念冥想共 8 次课，每次约 2 小时，由取得资格证的专业人员指导。探究正念冥想对游戏成瘾干预效果，以及这一过程中伴随的认知机制。

The efficacy of MM on IGD and its relevant brain mechanisms.

The current study aimed to investigate the efficacy of MM in helping people with IGD and to explore the

实验程序(Procedure)

整体实验流程：

实验者需要参与正念冥想的全过程。正念冥想共 8 次课，由有资格证的导师带来进行。

实验前后进行认知任务测查，具体要求依据电脑上呈现的字符做出按键反应。在参与者反应过程中，通过功能磁共振记录受试者大脑活动特征。

Please see this in the above protocol section.

费用(Costs)

本研究不会向您或您的医疗保险收取任何费用。(No charges will be billed to your insurance company or to you for this study.)

潜在风险和副作用(Risks and Side effects)

除去那些身上装有电子的、磁的或机械的设备（如心脏起博器）的人，或有颅内动脉夹者，已知的磁共振成像对人的危害或副作用非常小。最大的风险是受到磁场吸引的金属物体在其飞行运动中击中您。为了减少这种风险，我们要求所有参与研究的人在进入磁体间之前除去身上所有的金属物品。高速 MRI 无任何已知的风险。本研究将使用的扫描序列和扫描参数都是经美国食品和药品管理局审核并确认安全的。

磁共振扫描不会产生不适，但若您对置封闭空间感到恐惧，请告诉实验主试。在实验中，您会听到类似敲击的声音，佩戴耳塞后，这些声音没什么影响。

在实验中保持头部静止对本研究非常重要。用来固定头部的填充物一般感觉比较舒适，但您如果感到不适，请告诉实验主试。您可以在实验的任何时候无需任何理由终止实验。

本研究进行的扫描成像不是为了医学诊断。但是，如果实验者在实验中发现您的图像有可疑的异常，他们会建议您去看相关的医生。

All participants completed a safety screening questionnaire for fMRI. Individuals were excluded for the following: (1) Any non-gaming mental or neurological diseases or related histories; (2) Cognitive impairment on the MINI (Yves Lecrubier et al., 1997; Sheehan et al., 1998) or depression on the Beck Depression Scale (Beck et al., 1967); (3) Surgery, head trauma, or heart-related

diseases in the past year; (4) Claustrophobia; (5) Metal implants and tattoos of the neck or head; (6) Any SUDs during the last 12 months before recruitment; (7) Use of any psychotropic medication regularly; and, (8) Any prior mindfulness training experiences.

补偿(Compensate)

本临床试验如发生与试验相关的损害，医院将根据损害程度，依据国家相关法律、法规进行赔偿。（In this clinical trial, if there is any damage related to the trial, the hospital will compensate according to the relevant national laws and regulations.）

隐私(Confidentiality)

本研究的结果可能会在学术期刊/书籍上发表，或者用于教学。但是您的名字或者其他可以确认您的信息将不会在任何发表或教学的材料中出现，除非得到您的允许。另外，在本研究过程中取得的能够确认您身份的照片、录音或者录像，都将在得到您的书面允许之后才会使用。

参与实验人群具有随时退出实验的权力，且不需要提供任何退出的原因。
Participants have the right to stop or quit, and this selection without any excuse.

被试声明(Subject Statement)

我确认已经被告知本研究的目的、过程、可能的风险和副作用以及潜在的获益和费用。我的所有问题都已得到满意的回答。我已经详细阅读了本被试知情同意书。我下面的签名表明我愿意参加本研究。

签名(Signature): **日期(Date):**

主试声明(Experimenter Statement)

我已经解释了研究的目的、研究的程序、潜在的危险和不适以及被试的权益，并尽最大可能回答了与研究有关的问题.

签名(Signature): 日期(Date):
请您认真阅读并填写“被试知情同意书”和“被试检查单”，并注意**保留被试知情同意书**和**实验主试的联系方式**。<br>**实验费用由研究者承担。**(All of your expenses are undertaken by the researcher.)
