# Supplementary material for: Efficacy and Neural Mechanisms of Mindfulness Meditation Among Adults With Internet Gaming Disorder: A Randomized Clinical Trial
Source: JAMA Netw Open. 2024 Jun 18;7(6):e2416684. doi: 10.1001/jamanetworkopen.2024.16684 (PMC11185988; doi:10.1001/jamanetworkopen.2024.16684)
Supplement: Supplement 2. — eAppendix. Details for Mindfulness Meditation (MM) and Progressive Muscle Relaxation (PMR) Training Used in the Current Study eFigure 1. Timeline for Treatment and Assessments eFigure 2. Correlation Between Brain Response Changes in the Fusiform Gyrus (FG) and Craving Score Changes eFigure 3. Correlation Between Brain Response Changes in the Sublobar Region and Craving Score Changes eTable. Brain Responses Between MM and PMR Groups at Posttreatment and Pretreatment (Analysis of Variance) eFigure 4. Changes in Brain Responses Associated With MM and PMR eFigure 5. Regions of Interest Selected for Further Analysis eFigure 6. Functional Connectivity (FC) Changes in Medial Frontal Gyrus (MFG)-Lentiform Nuclei Mediate the Association Between Changes in Mindfulness and Craving Scores (Posttreatment to Pretreatment) in the MM Group [file jamanetwopen-e2416684-s002.pdf]

Supplementary Online Content

Ni H, Wang H, Ma X, et al. Efficacy and neural mechanisms of mindfulness meditation among adults with internet gaming disorder: a randomized clinical trial. *JAMA Netw Open*. 2024;7(6):e2416684. doi:10.1001/jamanetworkopen.2024.16684

eAppendix. Details for Mindfulness Meditation (MM) and Progressive Muscle Relaxation (PMR) Training Used in the Current Study

eFigure 1. Timeline for Treatment and Assessments

eFigure 2. Correlation Between Brain Response Changes in the Fusiform Gyrus (FG) and Craving Score Changes

eFigure 3. Correlation Between Brain Response Changes in the Sublobar Region and Craving Score Changes

eTable. Brain Responses Between MM and PMR Groups at Posttreatment and Pretreatment (Analysis of Variance)

eFigure 4. Changes in Brain Responses Associated With MM and PMR

eFigure 5. Regions of Interest Selected for Further Analysis

eFigure 6. Functional Connectivity (FC) Changes in Medial Frontal Gyrus (MFG)-Lentiform Nuclei Mediate the Association Between Changes in Mindfulness and Craving Scores (Posttreatment to Pretreatment) in the MM Group

This supplementary material has been provided by the authors to give readers additional information about their work.

© 2024 Ni H et al. *JAMA Network Open.*

eAppendix. Details for Mindfulness Meditation (MM) and Progressive Muscle Relaxation (PMR) Training Used in the Current Study

Details for MM training used in the current study (This English version is translated from Chinese. Please ask the authors for the Chinese version if desired.)

Lesson 1: Automatic Navigation	Introduction to the curriculum<br>Self-introductions<br>Group guidelines<br>Raisin Practice<br>Mindfulness exploration<br>Body scan practice
Lesson 2: Recognizing Triggers and Desires	Awareness of Breathing Practice<br>Homework review<br>Urge Surfing Meditation<br>Discussion on cravings and addiction
Lesson 3: Mindfulness in Daily Life	Sound awareness (opening)<br>Homework review<br>Mindful walking down the street<br>Self-care practice<br>Discussion on the inner critic<br>SOBER practice<br>Connecting with small mindful objects
Lesson 4: Dealing with Difficulties	Mindfulness meditation practice<br>Homework review<br>Nourishing and depleting<br>SOBER practice in challenging situations
Lesson 5: Acceptance and Skillful Responses	Mindfulness meditation practice<br>Mindful stretching<br>Homework review<br>SOBER practice in pairs<br>Wise responding<br>Discussion on acceptance
Lesson 6: Thoughts are just Thoughts	Awareness of thoughts<br>Homework review<br>Practice in classroom<br>ABCDEF model<br>Discussion on the relapse cycle
Lesson 7: Self-Care and Life Balance	Loving-kindness mindfulness practice<br>Homework review<br>Pebble meditation<br>Life tree discussion
Lesson 8: Continued Practice	Body scan practice<br>Homework review

© 2024 Ni H et al. *JAMA Network Open.*

	Resources and support network for continued practice<br>Course review and sharing<br>Stone meditation

Below are brief explanations of some of the mindfulness course contents:

Raisin Practice

This mindfulness practice involved eating a raisin mindfully. Participants were asked to explore the process of eating a raisin using their five senses: sight, touch, sound, smell, taste.

Urge Surfing Meditation

In urge surfing meditation, we asked participants to explore the thoughts, emotions and body sensations when an urge or carving arises and learn to co-exist with them. This may help participants to recognize the natural progression of urges that peak and diminish all on their own. While practicing, participants ride out an urge, like a surfer riding a wave.

SOBER Practice

There are five steps in this practice: Stop, Observe, Breathe, Expand, and React.
This practice can be short and used whenever participants want, especially when they feel they have tan urge or craving to play video games.

In the first two steps, participants can practice stopping and observing what is going on in this moment, including noticing emotions, body sensations and thoughts. In the third step, they can focus their attention on breathing, wherever they feel the breathing is most vividly experienced in their bodies In the fourth step, they may expand their attention to the entire body, noticing any sensation that appears in their bodies. In the last step, if they want, they can react with awareness, or just let it be.

Nourishing and Depleting

We start by introducing the exhaustion funnel: when we start to feel depressed or stressed, we tend to neglect the nourishing activities which usually help us feel better, and try to keep doing those which we really have to do – which may further deplete and exhaust us. We then feel even worse, so do even less.

We then asked participants to write down a list of daily activities and to mark which activities are nourishing and which are depleting.

SOBER Practice in Pairs

In this practice, two participants were asked to discuss their recent problems or struggles. During this process, a facilitator will stop with a bell ring, and ask them to practice SOBER in that moment. This process can help people to use the SOBER practice in their daily lives.

Life Tree Discussion

We asked each participant to draw a tree of their life. In these trees, five areas were depicted: social, working, learning, mental and physical health, entertainment. Participants were asked to fill in what

© 2024 Ni H et al. *JAMA Network Open.*

kind of life they would like to have in each area, and were given permission to add any other type of activities to their life trees.

Stone Meditation

In the last session, participants were given a small stone as a mindful gift. Facilitators guided participants in a meditation to promote experiencing the stone with their five senses. With this connection to the stone, facilitators hoped the participants could use this small stone as a reminder of their mindfulness practices, and continue their practices after the final session.

Details for PMR used in the current study

Find a quiet place free from distractions. Lie on the floor or recline in a chair, loosen any tight clothing, and remove glasses or contacts. Rest your hands in your lap or on the arms of the chair. Take a few slow even breaths.

The progressive muscle relaxation exercise aims to train individuals to detect muscle tension and relax their muscles. By intentionally tensing and then relaxing various muscle groups, individuals learn to distinguish between tension and relaxation sensations. The exercise involves tensing and relaxing the fists, arms, shoulders, neck, jaw, facial muscles, chest, back, abdomen, gluteal muscles, legs, and so on. Throughout the exercise, individuals focus on their breathing and the feeling of relaxation in their body. The goal is to practice this exercise to enhance relaxation skills.

Here are specific instructions for the progressive muscle relaxation exercise：

Hello, I will guide you through a progressive muscle relaxation exercise which aims to train you on how to detect tension and how to relax your muscles. During the process, you will intentionally tense various groups of muscles, notice the feeling of tension, and intentionally relax those muscles. Through repeated practice, you will learn to distinguish between muscle tension and relaxation sensations.

You can sit on a comfortable chair with both feet on the ground or lie down on a bed and your eyes. Follow the instructions provided.

When you tense your body muscles, please keep the tension for five to eight seconds, and then relax. If you need to practice muscles that were previously injured, please inform your doctor beforehand and avoid exerting too much force to prevent muscle strains.

Let's get started.

First, we need to get into a comfortable position to relax. Focus your attention on your breathing and feel the airflow within your body. Feel yourself in a comfortable resting position. Which parts of your body are touching the chair or bed? Think about your fingers and gently wiggle them.

Now, clench your right fist tightly and tense it further. Feel the tension in your hand. Try to pay attention only to the right fist and keep the rest of your body muscles relaxed. Sense the feeling of tension. Keep holding your fist tightly. Now, relax your fist and feel the difference between the relaxed tense sensations.

Repeat the same process with your right fist. Understand that tense and relaxed sensations are totally opposite. Now, tense up and then relax your right fist. Feel the difference between the tense and relaxed sensations in your right hand.

Next, clench your left fist and tense it further. Feel the tension in your hand, then relax it. Compare the difference between the relaxed and tense sensations.

© 2024 Ni H et al. *JAMA Network Open.*

Now, clench both fists together tightly. Feel the tension in your hands and forearms. Focus your attention on both fists and keep holding them tightly. Then, relax them and feel the difference between the tense and relaxed sensations.

Bend your elbows and flex your biceps strongly. Try to make your biceps as tense as possible. Feel the tension in this muscle and observe the tension in your upper arms. Then, relax your biceps and straighten your arms. Feel the difference between the relaxed and tense sensations.

Now, lift your shoulders up as high as possible and feel the sensation of almost touching your ears. Feel the tension in your raised shoulders. Emotions can often make our shoulders tense. Now, relax your shoulders and let them drop down to the most comfortable and relaxed.

Try to raise your shoulders backward and feel the tension. Then, relax them and feel the relaxation in your neck, throat and shoulders. This relaxation is straightforward, so just let it deepen slowly.

Now, tilt your head back as far as possible and feel the tension in your neck. Tighten the muscles of your neck, but don't use too much force. Turn your head slowly to the right and feel the change in muscle tension. Then, turn your head slowly to the left. Now, lower your head and touch your chin to your chest. Feel the tension in the back of your neck and throat. Relax and bring your head back to the most comfortable and relaxed position.

After you have relaxed the muscles around your neck, you should deepen that feeling of relaxation. Now, bite down hard. Close your jaw tightly and feel the tension in your jaw. Tight jaw muscles often indicate anxiety. Feel your upper and lower teeth in mouth simultaneously. Hold the tension for a while, then relax, and let your chin open a little.

Close your eyes lightly. Now, open your mouth as wide as possible. Hold the tension for a while, then relax and let your chin drop down. Experience the difference between tension and relaxation.

Make your tongue rise to the roof of your mouth. Make the top of your throat ache a little. Relax your tongue and lips, make a lip-tight circle and feel the tension in your mouth. Hold the tension for a while, then relax your lips. Release all the tension in your facial muscles, including your forehead, scalp, eyes, chin, tongue, and lips.

Focus on your forehead. Now, squint your eyes hard and wrinkle the muscles on your forehead. Feel the tension in your forehead. Now relax yourself. Imagine your whole forehead and scalp becoming more and more peaceful, then feel the difference when your forehead is completely relaxed.

Frown as hard as you can, feel the tension in your facial muscles, hold the tension for a while, and then relax. Imagine your face returning to a state.

Now, close your eyes tightly, and feel the tension in your eye muscles. Hold the tension for a while, then relax your and gently rub your eyes. Experience the difference between tension and relaxation.

Now, take a deep breath and exhale all the air from your lungs. Your body will naturally inhale air, filling your lungs. Fill the space in your lungs with air by inhaling again. Hold your breath after inhaling. Hold your breath to allow your lungs to be fully with air. Feel the tension in your chest now. Hold your breath for a while. Pay close attention to this tension, and then release it. Gently exhale, letting the air flow out of your mouth.

Continue to relax. Now, let your body breathe autonomously, in a gentle and relaxed manner. Yes, your body knows how to breathe. Notice that you feel comfortable when your lungs are full of air and you are holding your breath. Be aware of the sensation of tension. Then exhale, letting the air flow out of your mouth. Imagine that all the tension in your body is being exhaled with this breath.

Now arch your back, but be careful not to strain your muscles. Let the rest of your body relax as much as possible. Focus on the arching of your back while arched. Feel this tension. Now, relax. Release all the

© 2024 Ni H et al. *JAMA Network Open.*

tension. Relax deeply. Then, tighten your abdominal muscles. Maintain this tension, and then exert even more force. Note this sensation tension. Then release it.

Now place your hands on your abdomen. Expand your belly and take a deep breath. Raise your hands. The deeper you breathe, the higher your hands will be. Hold your breath when your hands are at the highest point. Use your expanded belly to hold your hands in place. Maintain this position for a moment, and then let go. As you exhale, feel the sensation of relaxation. Remember, abdominal muscles are relaxed as you exhale. Notice the difference between tension and relaxation.

Now, tighten your gluteal muscles. Let the rest of your body relax as much as possible. Maintain this tension, and then exert even more force. Note this sensation of tension. Then release it.

Now, lift your stretched legs fifteen to twenty centimeters high, causing the tension in your thigh muscles. Now relax. Place your legs back on the ground. Note the difference between tension and relaxation.

Continue to stretch your legs. Now, curl your toes and the top of your foot upward, dorsiflexing, contracting the neck of your anterior calf as much as possible. Try to lift the top of your foot upward. Feel this tension. Now, relax. Feel the emergence of a relaxed feeling. Relax. Relax.

Now, let your toes point downward, contracting your calf muscle of the posterior compartment of your lower leg. Try to point your toes downward as much as possible. Note the tension in your toes and calf muscles. Now relax. Release all the tension. Note the difference between tension and relaxation.

Relax all other muscles in your lower body. Finally, focus on your toes. Keep your toes straight and curled. Maintain this tension. Note this sensation without excessively pulling up other muscles. Then relax.

Now, let your toes point upward. Maintain this tension. Then relax. Allow this relaxation to deepen. Feel the weight of your lower body. Focus your attention on relaxing your feet, ankles, anterior and posterior calf muscles, knees thighs, and buttocks. Feel the spread of relaxation throughout the lower abdomen, lower back, upper back, and chest. Let the feeling of relaxation become more profound. Are there any other muscles in your body that need to be relaxed? Feel this sense of relaxation spread to your shoulders, arms, palms, fingers. Feel your neck, chin, and eyes, and all your facial muscles become soft and calm. Feel the distribution of hair on your scalp. Let the warm and comfort on the top of your head. Observe your relaxed body. Recognize the difference between tension and relaxation. Breathe slowly, relax. Release all the tension in your body. Relax and let your body stay calm. Remember the relaxation.

Similarities and difference between MM and PMR

Regarding similarities, both MM and PMR benefit from quiet places and sitting or lying down. Both focus on some bodily sensations.

Regarding differences, while PMR focuses on the relaxation of muscles, MM not only asks participants to relax themselves in their bodies, but also asks them to do work in the mind.

Behavioral results

Regarding IAT scores, a significant group (MM, PMR) * time (pre-, post-treatment) effect was observed ($p<0.001$). Further analyses revealed that in the MM group, the IAT score decreased significantly at post-treatment (pre (mean [SD], 70.09 [10.06]); post (mean [SD], 43.00 [12.02]))($p<0.001$). IAT scores did not change significantly in the PMR group (pre (mean [SD], 69.34 [9.72]); post (mean [SD], 63.22 [10.70])) ($p=0.111$). At post-test, the MM-related decrease was more substantial than that in the PMR group ($p<0.001$) (Figure 2B in the main text).

© 2024 Ni H et al. *JAMA Network Open.*

Regarding craving scores, a significant group (MM, PMR) * time (pre-, post-treatment) effect was observed ($p<0.001$). Further analyses revealed that in the MM group, the craving score decreased significantly at post-treatment (pre (mean [SD], 58.78 [15.68]); post (mean [SD], 33.63 [12.02])) ($p<0.001$). Craving scores did not change significantly in the PMR group (pre (mean [SD], 57.36 [12.12]); post (mean [SD], 50.86 [10.96])) ($p=0.144$). At post-test, the MM-related decrease was more substantial than that in the PMR group ($p<0.001$) (Figure 2C in the main text).

© 2024 Ni H et al. *JAMA Network Open.*

eFigure 1. Timeline for Treatment and Assessments

	Pre-test	Time1	Time2	Time3	Time4	Time5	Time6	Time7	Time8	Post-test
Safety assessments	◆									◆
Groups										
MM		◆	◆	◆	◆	◆	◆	◆	◆	
PMR		◆	◆	◆	◆	◆	◆	◆	◆	
Data collection										
DSM-5	◆									◆
IAT	◆									◆
fMRI: Cue	◆									◆
fMRI: Rest state	◆									◆
fMRI: T1	◆									◆
Craving (QGU)	◆	◆	◆	◆	◆	◆	◆	◆	◆	◆
FFMQ	◆									◆
	Baseline	Treatment								Outcome

A

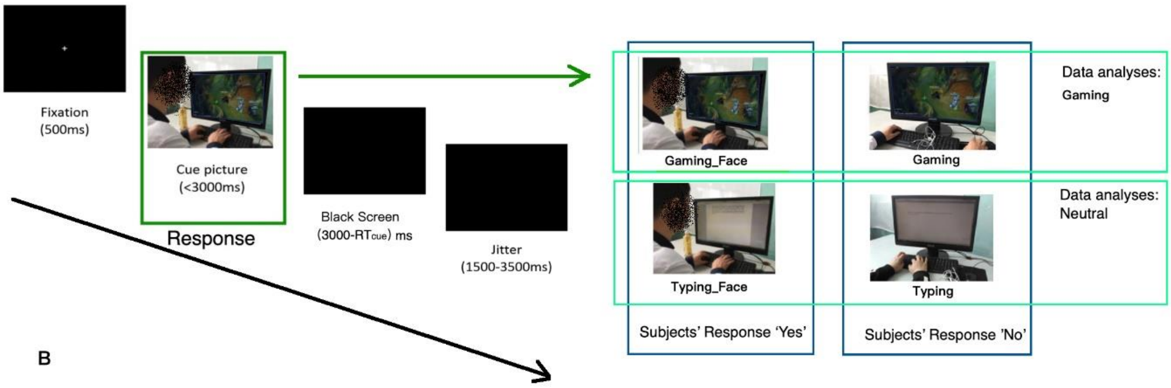

A, Randomization of participants into groups and the detailed arrangement for mindfulness meditation (MM) and progressive muscle relaxation (PMR) training. B, The cue-craving task used during pretreatment and posttreatment (the faces in the figure were masked for publication to obscure potentially identifying information). *DSM-5* indicates *Diagnostic and Statistical Manual of Mental Disorders, Fifth Edition* proposed criteria for IGD; FFMQ, Five Facet Mindfulness Questionnaire; IAT, Internet Addiction Test (revised version for IGD); IGD, internet gaming disorder; QGU, Questionnaire for Gaming Urges (adapted for IGD from the Tiffany Questionnaire for Smoking Urges).

© 2024 Ni H et al. *JAMA Network Open.*

Task-based brain responses

eFigure 2. Correlation Between Brain Response Changes in the Fusiform Gyrus (FG) and Craving Score Changes

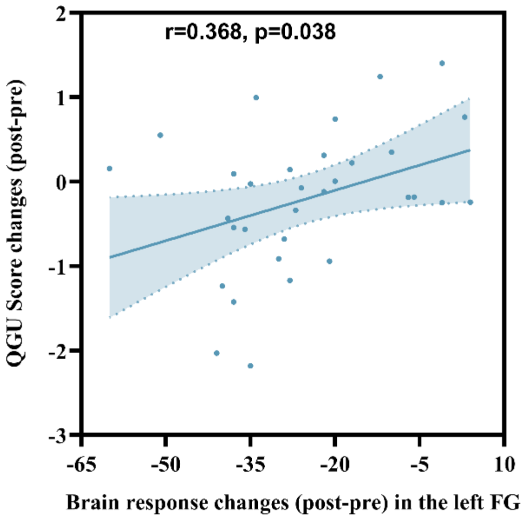

eFigure 3. Correlation Between Brain Response Changes in the Sublobar Region and Craving Score Changes

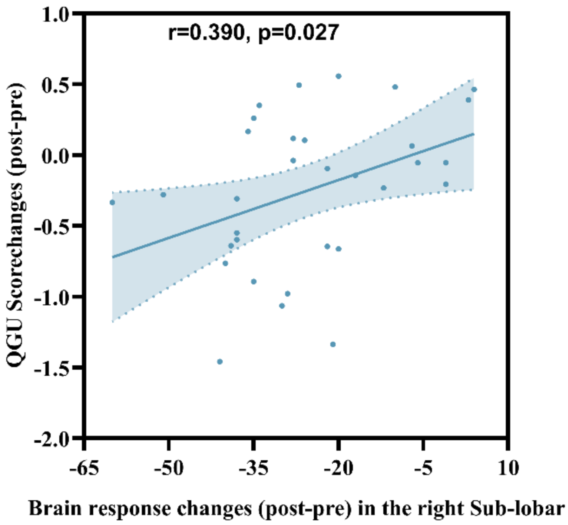

© 2024 Ni H et al. *JAMA Network Open.*

eTable. Brain Responses Between MM and PMR Groups at Posttreatment and Pretreatment (Analysis of Variance)

Cluster Number	x,y,z [a]	Peak Intensity	Cluster size [b]	Region [c]	Brodmann's Area
1	-21,-3,-12	-5.33	68	L lentiform Nuclei	
2	18,-6,-9	-4.735	54	R Lentiform Nuclei	
3	27,-27,-3	-5.101	43	R Insula	
4	-12,48,3	-4.668	142	R Medial Frontal Gyrus	10
5	-9,0,-18	-5.218	226	L Sub-lobar	

[a] Peak MNI Coordinates.

[b] Number of voxels

[c] The brain regions were referenced to the software Xjview (http://www.alivelearn.net/xjview8).

© 2024 Ni H et al. *JAMA Network Open.*

eFigure 4. Changes in Brain Responses Associated With MM and PMR

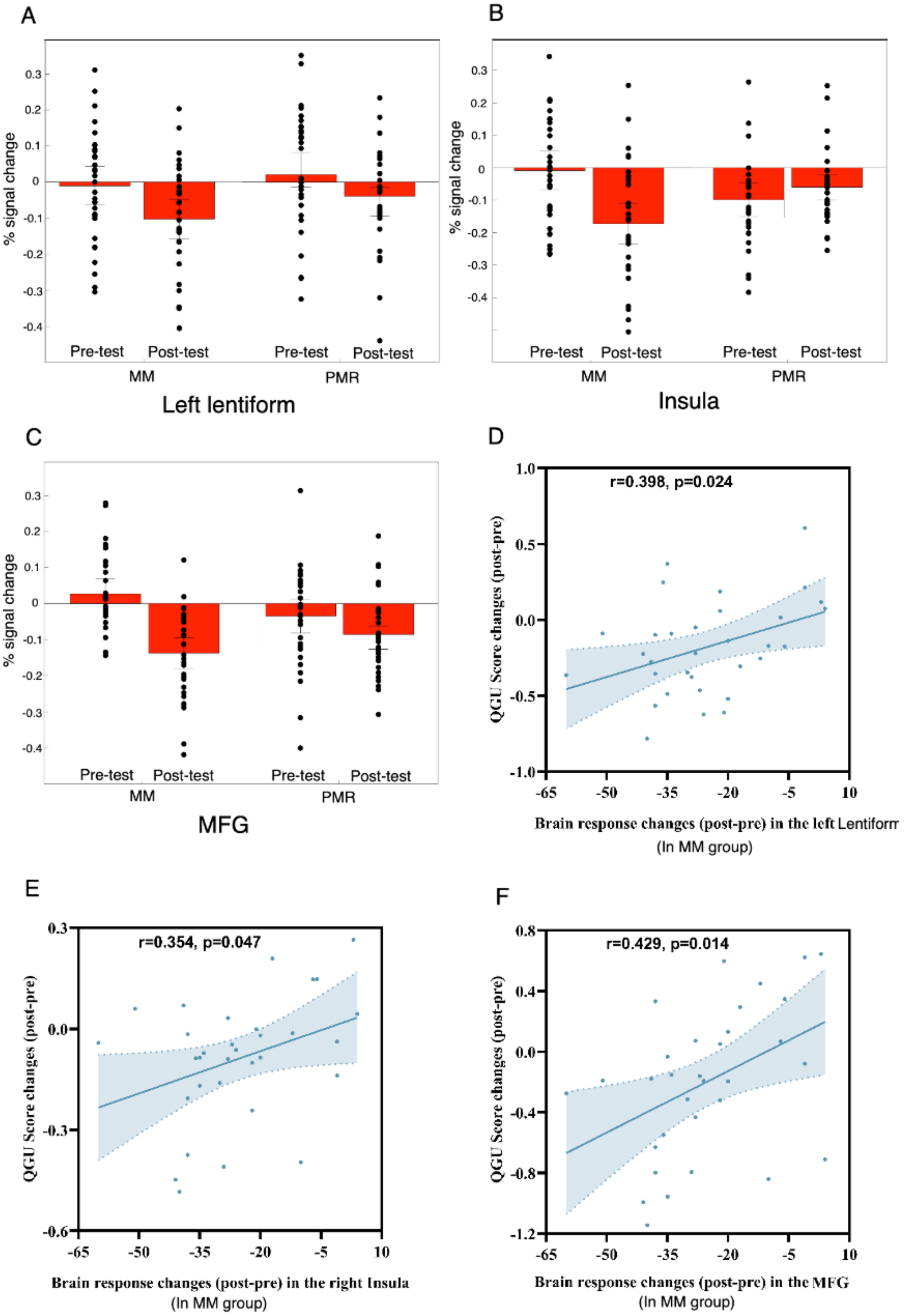

(A, B, C) Changes in brain activity (beta weights) in the surviving brain regions in the left lentiform, right insula, and left MFG from pre- to post-treatment in the MM and PMR groups; (D, E, F) Correlations between changes in brain responses in the lentiform, insula and MFG and the changes in craving scores in MM subjects. The dotted horizontal lines in A,B,C were 95% CIs.

© 2024 Ni H et al. *JAMA Network Open.*

eFigure 5. Regions of Interest Selected for Further Analysis

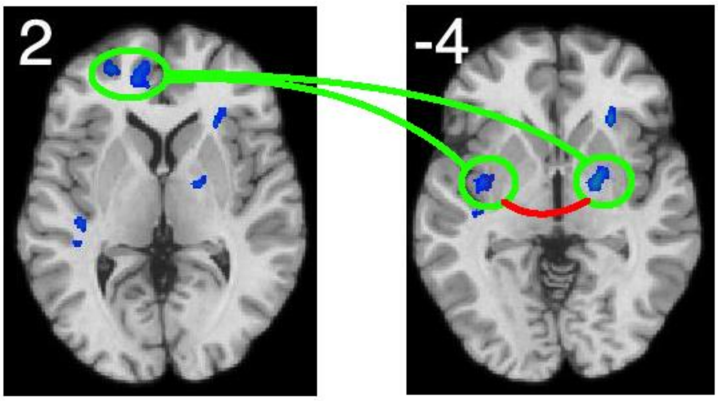

Mediation analysis

In the MM group, changes in MFG-Lentiform FC were correlated with changes in gaming craving scores (r=-0.246, p=0.023) and mindfulness scores (r = 0.231, p =0.021). Based on these findings and the hypothesis that changes in mindfulness would operate through specific brain mechanisms to reduce craving, we performed a mediation analysis using the changes in MFG-Lentiform FC as the mediating factor. We found that the changes in MFG-Lentiform FC mediated the relationship between the changes in MM and gaming craving scores in the MM group (see Supplementary eFigure 6).

© 2024 Ni H et al. *JAMA Network Open.*

eFigure 6. Functional Connectivity (FC) Changes in Medial Frontal Gyrus (MFG)-Lentiform Nuclei Mediate the Association Between Changes in Mindfulness and Craving Scores (Posttreatment to Pretreatment) in the MM Group

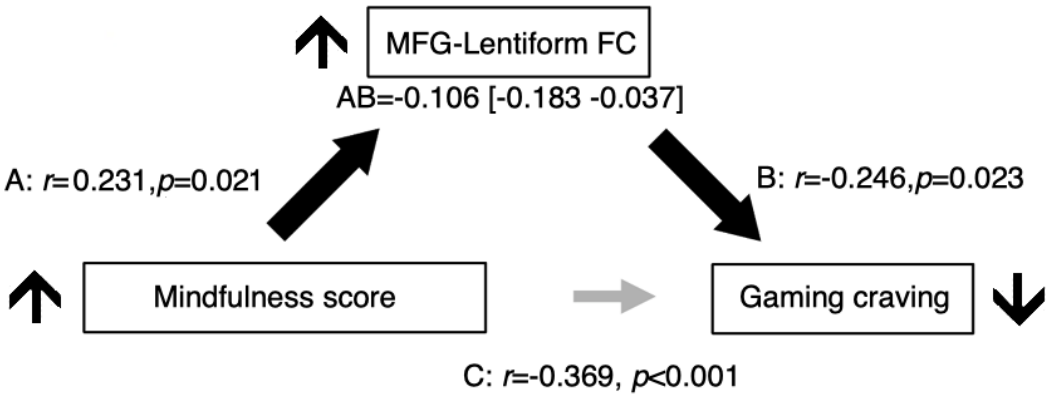

© 2024 Ni H et al. *JAMA Network Open.*
