# Supplementary material for: Efficacy and Neural Mechanisms of Mindfulness Meditation Among Adults With Internet Gaming Disorder: A Randomized Clinical Trial
Source: JAMA Netw Open. 2024 Jun 18;7(6):e2416684. doi: 10.1001/jamanetworkopen.2024.16684 (PMC11185988; doi:10.1001/jamanetworkopen.2024.16684)
Supplement: Supplement 3. — Data Sharing Statement [file jamanetwopen-e2416684-s003.pdf]

Data Sharing Statement

Ni. Efficacy and Neural Mechanisms of Mindfulness Meditation Among Adults With Internet Gaming Disorder. *JAMA Netw Open*. Published June 18, 2024.
doi:10.1001/jamanetworkopen.2024.16684

Data
Data available: No

Additional Information
Explanation for why data not available: Data availability The data stored at our lab-based network attachment system: http://QuickConnect.cn/others. ID: guests; PIN dong@123.COM.
